# A Theoretical Approximation of the Accelerating Effects of Ultrasound about the Extraction of Phenolic Compounds from Wood by Wine Spirits

**DOI:** 10.3390/foods11040517

**Published:** 2022-02-11

**Authors:** Manuel J. Delgado-González, María de Valme García-Moreno, Dominico A. Guillén-Sánchez

**Affiliations:** Departamento de Química Analítica, Facultad de Ciencias, Instituto de Investigación Vitivinícola y Agroalimentaria (IVAGRO), Campus Universitario de Puerto Real, 11510 Cádiz, Spain; manuel.delgado@uca.es (M.J.D.-G.); dominico.guillen@uca.es (D.A.G.-S.)

**Keywords:** kinetics, extraction, phenols, wood, spirits, aging

## Abstract

The acceleration on the extraction by the sonication of phenolic compounds (measured as the Total Phenolic Index) from wood chips by wine distillates is studied in the present paper. Using the Arrhenius equation, the theoretical temperature at which the kinetics obtained by these sonicated extraction processes are equal to the kinetics of non-sonicated and thermally accelerated extractions, was calculated. By applying a pseudo-second order kinetic model, it was shown that the initial rate values obtained from the sonicated extractions were as high as those obtained from the thermal extractions carried out at a temperature at least 2.5 °C higher than the real temperature at which the experiment was performed. Higher power densities lead to higher initial rates of extraction, although very high power densities decrease the amount of phenols in equilibrium, probably due to the degradation processes. Additionally, the positive synergy between the sonication and the movement of the recirculated distillate through wood chips was also stablished, obtaining a difference of temperature of at least,18.2 °C for the initial extraction rate and 7.0 °C for the equilibrium.

## 1. Introduction

Aging is an important phase in the production of many different spirits, such as rum, whisky or brandy. During the aging process, the alcoholic distillates extract all the compounds from wood, which are characteristic of the desired final product [1,2]. There are many compounds that are extracted by the distillates from the wood in aging processes (such as volatile esters, colored compounds, organic acids and phenols). Phenolics, which are a family of organic molecules that comprises more than eight thousand known substances [3], are considered as the main compounds extracted from wood due to their importance and their predominance in aged beverages. For this reason, the phenolic compounds’ content (usually measured as the Total Phenolic Index or TPI) is a crucial factor in beverage aging, and it has been extensively used as a marker for such processes [4,5]. The extraction of phenolics from wood is a complex process, which can be classified according to two different types of mechanisms: physical [6,7] and chemical [8,9,10].

The physical mechanisms are based on the direct solvation of the substances that are present in the wood [6], such as the hydrolysable tannins, which are the most abundant compounds transferred into aged spirits [7]. These physical processes are usually formed by two steps: the first one consists of rapidly washing off the molecules from the surface of the wood, while the second step consists of the slow extraction of the phenolic compounds as a result of the diffusive movements of the distillate passing through the wood pores [11].

On the other hand, in aging processes, numerous and complex chemical reactions take place. This was already stated by Puech [8], who reported that some phenolic compounds are obtained through a complex procedure that involves the extraction and oxidation of the lignin that is found in wood casks. Moreover, it should be noted that hydrolysable tannins may suffer specific degradation processes due to the oxidative alcoholic media, which transforms them into ellagic and gallic acid molecules [10]. In addition, not only the compounds that are on the surface of the wood are involved in the extraction, but also some of the substances that are within the distillate [9]. 

As these extraction mechanisms are usually quite slow, aging procedures may take several months, even years to return a desirable product. This way, the different researches that intend to develop new aged alcoholic products are costly and require a considerable amount of time. Therefore, several accelerated extraction systems that intend to provide producers with an approximate notion of their final product features, although in a shorter length of time, were studied. It has been widely established that the extraction rates of the phenolic compounds increase with the increase in temperature [12,13]. There are several factors associated with temperature, which accelerate the extraction processes: firstly, the higher thermal energy of the molecules in the solvent raises the probability of collision between the distillate molecules and the phenols, and thus overcomes the necessary activation energy and breaks the bonds between the phenolic compounds and the solid (wood) [14]; secondly, when the temperature of the system is increased, the solvent viscosity decreases and the wood pores expand. Consequently, the flow of the molecules in the solvent through the wood pores is also favored and, in turn, accelerates the diffusive extraction of the phenolic compounds that are present in the solid [15,16]. In addition, higher temperatures also increase the solubility of the phenolic compounds in the distillate according to Van’t Hoff equations used for precipitation and solubility equilibriums [17]. On the other hand, the thermal degradation of phenols is a widely studied phenomenon, since they have been found to be destabilized at high temperatures [18]. 

One of the most innovative methods to accelerate phenolic extraction over beverage aging processes is based on the application of ultrasound to the extraction system. In this sense, our research team developed and studied an previous accelerated beverage aging system based on the continuous flow of alcoholic distillates from a deposit to a sonicated extraction cell filled with wood chips [4]. Ultrasounds (US) with frequencies between 20 kHz and 10 MHz were frequently used to accelerate the extraction of compounds from organic solids into liquid media [19], since it causes the cavitation phenomenon, which breaks the cells’ tissues. Cavitation is triggered by high-frequency sound waves applied to a liquid with elastic properties; sound waves cause the expansion of the liquid molecules generating cavities that can collapse in an implosive manner. This phenomenon releases a large amount of energy (it was found to return an effective temperature of 5075 K) into the extraction media and generates localized pressure spots that break the vegetable tissues and favor the extraction of the vegetable compounds by the liquid medium. In addition, the sonication of water produces oxidative species (hydroxyl radical and hydrogen peroxide) [20], which may favor the oxidation of the lignin and the degradation of the hydrolysable tannins that are obtained from wood. 

Wood is not a homogenous solid, since it presents a heterogeneous porous surface [21]. As a result of this fact, the kinetics of the extraction mechanisms and the diffusion of phenolic compounds from the wood to the distillate are complex and not easily explained. This way, in order to reach an approximate description of the extraction kinetics of the process, different kinetic models had to be applied. The kinetics of the extraction of phenolics from different solid matrixes were published by several research studies [11,22,23,24]. In those works, the most often used mathematical models applied to the study of the extraction kinetics of phenolic compounds are the Lagergren logarithmic first order model and the Peleg’s hyperbolic pseudo-second order model. 

The first order model was applied, for instance, to study solid–liquid extractions from tilia sapwood [22], and it was reported to fit better with the initial stages of most solid–liquid extraction processes [25]. Additionally, as it was explained by Covelo et al. [26], the Lagergren model better explains the extraction procedures that are controlled by one fast process, such as the physical absorption of the soluble substances, which are bound onto the surface of the solid. On the other hand, the pseudo-second order kinetic model has been widely used to explain different solid–liquid extractions [27]. It fits better with procedures that can be divided into two steps: e.g., a first and quick step that includes the physical absorption of the soluble molecules from the first layers of the solid, and a second and slower step, which is controlled by the physical diffusive mechanisms or chemical reactions that extract molecules from within the solid [26]. Better results are usually obtained from this latter pseudo-second order model. In this sense, Psarra et al. [11] confirmed that Peleg’s model returned coefficients of determination (R^2^) that were higher than 0.98, which explained the extraction of the phenolic compounds from different types of wood chips by wine model solutions. In addition, the effect on the kinetic properties resulting from the application of ultrasounds were also studied [16,28], and the successful acceleration of the extraction process of the phenolic compounds by sonication has been extensively proven in the related literature.

This paper intends to go one step further on the study of the application of ultrasounds to accelerate spirit aging, by comparing the effect of the ultrasound energy with the accelerative effect of higher temperatures on these kinds of processes. For this purpose, two different types of phenolic extractions of spirits from wood were carried out: thermal extractions at different temperatures, but without the sonication of the media, and ultrasound-assisted extractions at 20 °C. Then, the kinetics corresponding to both types of extractions were equated by means of the Arrhenius equation. This way, we could comprehend the magnitude of the acceleration produced by ultrasounds, by obtaining the theoretical temperature at which a particular thermal extraction must be performed to obtain the same extraction rates that we would obtain by the sonication of the media. In addition, the effect on the extraction kinetics resulting from the application of ultrasound waves at three power density levels was studied. Moreover, following the same accelerated aging system that our team previously developed [4], we considered that it would be interesting to study the synergic effect on the kinetics of the extraction process that may result from not only the sonication, but also the movement of the distillate through the wood.

## 2. Materials and Methods

### 2.1. Samples

A wine distillate with an alcoholic strength of 65% of Alcohol By Volume (ABV), supplied by a winery associated with the Protected Designation of Origin of Jerez-Xérèz-Sherry y Manzanilla de Sanlucar de Barrameda was used for the extractions. These distillates were diluted down to 40% ABV. The American oak (*Quercus alba*) wood chips for the experiments were supplied by Nutritec (Barcelona, Spain). They were medium-sized (64 cm^2^·L^−1^) and were submitted to a medium toasting treatment for 5 min in an oven at 130–140 °C, according to the supplier. 

### 2.2. Total Phenolic Index

The TPI was determined by measuring the absorbance at 280 nm, using an UV-VIS spectrophotometer Agilent Cary-60 (Agilent, Little Falls, DE, USA) [29]. Gallic acid was used to generate the calibration curve, and the results were expressed in mg Gallic Acid Equivalent (GAE) per L of sample. The measurements were carried out in triplicate for the calibration curve and for the sample analyses. In the continuous flow analyses, the 280 nm measurements were carried out with a delay of 1 s, so that the sample could be renovated into the flow cell.

### 2.3. Extraction Procedures

The extraction experiments were carried out for 60 min under different conditions, including some non-sonicated extractions carried out at different temperatures (NUS samples), as well as pumped and non-pumped extractions carried out under different ultrasound power densities (US + M and US-M samples, respectively). The power density was used as a variable in this paper, because its usefulness for defining extraction procedures [30] has been previously established. Each of these experiments was carried out in triplicate.

**Figure 1:**
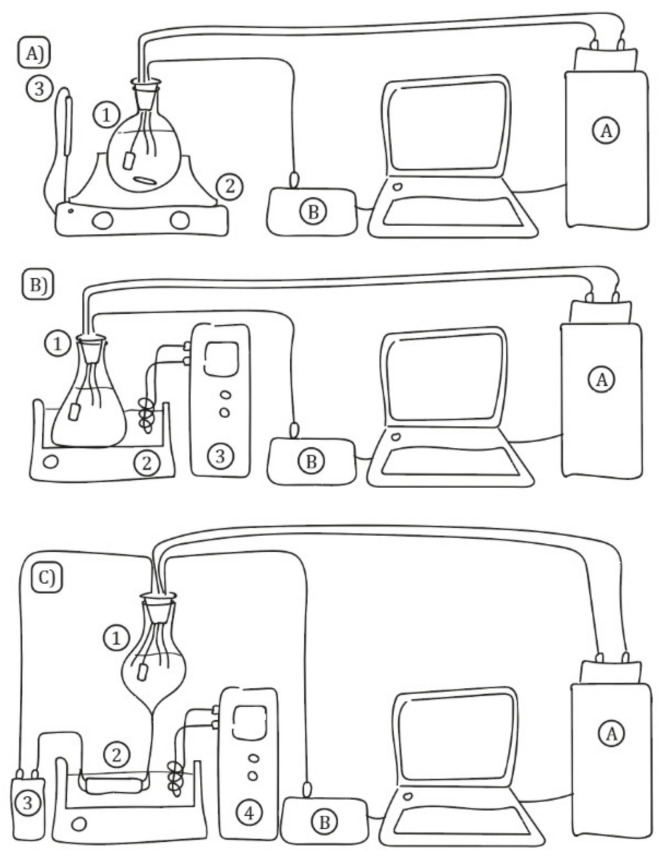
Laboratory systems. (**A**) Non-sonicated extractions at different temperatures (1: extraction cell, 2: heating plate and 3: thermocouple); (**B**) non-pumped sonicated extractions at different power densities (1: extraction cell, 2: ultrasound bath and 3: temperature controller); and (**C**) pumped sonicated extractions at different power densities (1: glass tank, 2: extraction cell, 3: pump and 4: temperature controller). In all diagrams: A: spectrophotometer and B: temperature sensor.

For the extractions that were carried out at different temperatures without sonication (Figure 1A), 1.1 g of wood chips was submerged into 250 mL of distillate in a round-bottom flask (1), which was previously thermostated at the desired temperature by means of a heating plate (2) RTC-basic (IKA, Staufen, Germany) fitted with a thermocouple (3) ETS-D4 (IKA, Staufen, Germany). Those quantities were chosen because they ensured the same surface/volume ratio as the aging in wood casks that are commonly carried out in our region. The mixture was continuously agitated at 300 RPM. The six studied temperature levels were (A) 20 °C, (B) 25 °C, (C) 30 °C, (D) 35 °C, (E) 40 °C and (F) 45 °C. On the other hand, due to the heat losses of the system and the variability produced by the heating plate, the real temperatures measured at the laboratory were (A) 19.56 °C, (B) 23.98 °C, (C) 31.27 °C, (D) 36.04 °C, (E) 38.34 °C and (F) 45.01 °C. This range of temperatures was restricted by the instability of the phenolic compounds at higher temperatures [18]. For the means of the homogenization of the temperature, the liquid was agitated at 50 rpm.

For the non-pumped extractions that were carried out at different ultrasound power density levels (Figure 1B), 1.1 g of wood chips was introduced into 250 mL of distillate in an Erlenmeyer’s flask (1) submerged into a 20 L ultrasound bath (2) P20 (J.P. Selecta, Abrera, Spain), which was thermostated at 20 °C by a temperature controller (3) F12 (Julabo, Seelbach, Germany). Due to the effect of the sonication of the water and the heat losses of the system, the actual temperature of the media measured in the laboratory were (A) 22.57 °C, (B) 22.07 °C and (C) 23.75 °C. As the ultrasound bath produced waves at 40 KHz with a fixed total power of 1000 W, the different power densities were controlled by changing the volume of the water inside the ultrasound bath: (A) 20 L for 50 W·L^−1^, (B) 15 L for 67 W·L^−1^ and (C) 10 L for 100 W·L^−1^.

For the pumped extractions that were carried out at different ultrasound power densities (Figure 1C), a glass tank filled with 250 mL of distillate (1) was connected by silicone tubes to a glass recipient filled with 1.1 g of wood chips, which was submerged into a 20 L ultrasound bath (2) P20 (J.P. Selecta, Abrera, Spain). An Aquarius Universal liquid pump (3) 2000 (OASE GmbH, Hörstel, Germany) was used to drive a 50 L·min^−1^ continuous flow of the distillate through the system. The ultrasound bath was thermostated at 20 °C by means of a temperature controller (4) F12 (Julabo, Seelbach, Germany). The real temperatures measured in the laboratory were (A) 25.15 °C, (B) 27.46 °C and (C) 26.55 °C. The different power density levels, namely, (A) 50 W·L^−1^, (B) 67 W·L^−1^ and (C) 100 W·L^−1^), were controlled by modifying the volume of the water inside the bath, as explained above.

For measuring the TPI, the absorbance at 280 nm of all the samples was registered online at 30 s intervals using a peristaltic pump Minipuls 3 (Gilson, Middleton, WI, USA), a quartz flow injection cell, with a light path of 10 mm, and an UV-VIS spectrophotometer Agilent Cary-60 (Agilent, Little Falls, DE, USA). In addition, the temperature of all the mixtures was directly recorded every second by means of a previously calibrated Arduino temperature sensor DS18B20 submerged in the distillate (B).

### 2.4. Kinetic Study

In this paper, the exponential first-order model and the hyperbolic Peleg’s model were applied to determine the extraction kinetics of the phenolic compounds in the wood chips over the aging process of the wine distillate samples.

#### 2.4.1. Exponential First-Order Model

The first order model used in our study was first developed by Lagergren [25]. Its mathematical expression can be observed in Equation (1), where *X*(*t*) is the analyte concentration at a time, *t*, *X_eq_* is the analyte concentration at equilibrium and *K*_1_ is the first order rate constant.

(1) $$X\left(t\right)=X_{eq}\left(1-e^{-K_{1}\cdot t}\right)$$

#### 2.4.2. Peleg’s Hyperbolic Model

The pseudo-second order model (Equation (2)) used in our study was developed by Peleg to describe moisture sorption curves [31].

(2) $$X\left(t\right)=\frac{t}{1/K_{1}+t/K_{2}}$$

where *X*(*t*) is the analyte concentration at a time, *t*, *K*_1_ is a rate constant related to the initial rate (*V*_0_) of the process according to Equation (3), and *K*_2_ is another constant related to the analyte concentration at equilibrium (*X_eq_*), as determined by Equation (4).

(3) $$V_{0}=1/K_{1}$$

(4) $$X_{eq}=1/K_{2}$$

To carry out the modeling of the extraction process of the phenolic compounds from wood chips by an alcoholic distillate over an aging process, an assumption is required, i.e., the TPI registered during the evolution of the extract should be considered as the result of the extraction of pseudo-homogeneous phenolic substances with similar chemical–physical properties.

#### 2.4.3. Arrhenius Kinetic Equation

As it was explained by Arrhenius in 1889, kinetic constants vary with the temperature of the system following the empiric formula shown in Equation (5) [17].

(5) $$K\left(T\right)=A\cdot e^{\left(-E_{a}/RT\right)}$$

where *K* is the kinetic constant at temperature *T*, *A* is a frequency constant that indicates the frequency of the collisions between the particles that are reacting, *R* is the universal gas constant and *E_a_* is the activation energy of the process.

### 2.5. Statistical and Mathematical Analyses 

All the mathematical calculations were carried out by means of Matlab R2019b (MathWorks, Natick, MA, USA). The kinetic modeling of the extraction curves was carried out by applying the non-linear regression models from Equations (1) and (2). The comparisons of the means of the obtained TPI for each of the extraction processes carried out were made by applying the ANOVA tests at 95% of the confidence level.

### 2.6. Comparison between the Sonicated and Thermal Extractions 

In order to quantify the actual acceleration effect on the extraction process of the phenolic compounds caused by the sonication of the extraction media, the equation between the kinetics obtained from the extractions under the effects of ultrasound waves and the kinetics obtained from the thermal extractions were applied. This comparison study allowed us to identify the theoretical temperature at which the extraction without sonication returned the same kinetic constants as the extraction under the effect of ultrasound.

For an easier visualization of the entire process described in this paper, the steps can be summarized as follows: (A) from the two applied kinetic models, selecting the one that returns the highest R^2^ values to fit the TPI extraction curves; (B) quantifying the modeled kinetic parameters of the non-sonicated extractions at different temperatures and then applying the Arrhenius equation (Equation (5)) to linearize the kinetic constants, according to the different extraction temperature levels; (C) determining the modeled kinetic parameters of the sonicated extractions at different ultrasound power levels and then, by interpolating these constants on the linearized Arrhenius equations previously obtained, determining the theoretical temperature at which these extractions would have been achieved in non-sonicated systems; and, (D) finally, as it is shown in Equation (6), calculating the difference between the theoretical temperature (*T_THEOR_*) obtained from the previous step and the actual temperature at which the sonicated extraction was carried out (*T_REAL_*). This temperature difference, as a result of the sonication effect (Δ*T_US_*), was used as an index to quantify the acceleration of the extraction process caused by each of the three different ultrasound powers levels applied.

(6) $$\Delta T_{US}=T_{THEOR}-T_{REAL}$$

In addition, the synergic effect between the sonication of the extraction media at different ultrasound power levels and the movement of the distillate through the wood chips was quantified by also determining the kinetic parameters of this process and repeating steps (C) and (D), as above explained.

## 3. Results and Discussion

### 3.1. Selection of the Best Kinetic Method to Explain the Extraction Processes

In order to quantify the effective acceleration of the extraction process caused by sonication, it was first necessary to determine which one of the two kinetic models that had been applied better fitted the TPI extraction curves. 

**Figure 2:**
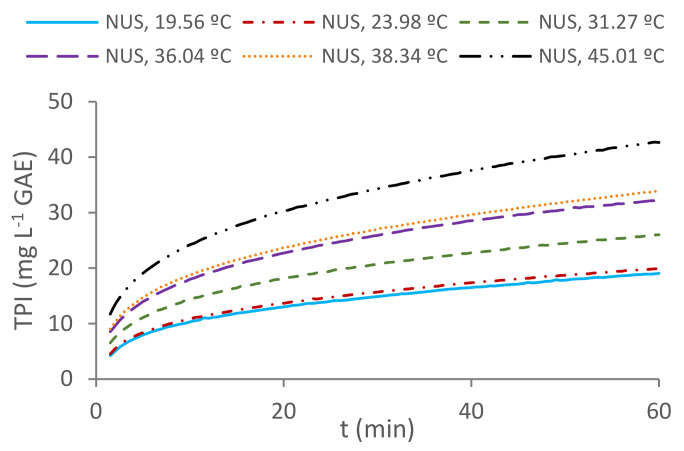
TPI vs. time plots of the non-sonicated extractions carried out at different temperatures (NUS).

The resulting TPI vs. time curves obtained by the six non-sonicated extraction experiments at different temperatures were plotted and can be observed in Figure 2. These curves show the typical extraction behavior at each of the temperature levels of the experiments; the TPI increment was rapid at the beginning of the process and slows down as the extraction continues. 

In relation with the temperature of the extraction media, higher extraction curves are obtained as the solvent’s temperature is increased. In fact, the TPI obtained after 60 min at 45.01 °C was 42.659 ± 0.469 mg·L^−1^ GAE, which doubles the TPI measured in the same time lapse of the extraction at 19.56 °C, with values reaching just 19.054 ± 0.229 mg·L^−1^ GAE. The accelerating extraction effect of an increasing temperature can be explained by the following factors: on one hand, the thermal energy contributes to break the solid–phenol bonds overcoming the activation energy barrier [14]; on the other hand, the combination of several phenomena, such as wood-swelling, faster solvent molecule movement through the wood pores and a lower viscosity at higher temperatures, favor diffusive extraction processes [11]. 

The R^2^ values obtained from each experiment when the studied kinetic models are applied (Equations (1) and (2)) are shown in Table 1. The Lagergren first order model obtained the worst coefficients of determination, with values lower than 0.938 in all the six temperature levels. On the other hand, Peleg’s pseudo second-order model fit the TPI vs. time curves better, with coefficients of determination of at least 0.987. It was previously stated in the literature that the Lagergren model explains better those sorption procedures, in which their extraction rates are affected by just a single rapid action. On the contrary, Peleg’s model better explains those sorption processes in which extraction rates are critically affected by two different processes, i.e., a rapid mechanism and a slower action [26]. In the case of the phenolic extractions from wood, the rapid action would be the instantaneous washing off, by the distillate fluid, of the phenols that are located on the wood surface. In addition, two slow mechanisms might explain why the Peleg’s model fits the extraction of the phenolic compounds present in wood better than the Lagergren model. One of them is the diffusive extraction of the phenols from the wood over a slow process that comprises three steps: (A) the penetration of the fluid into the wood; (B) the movement of the phenolic compounds within the wood and coming into contact with the fluid surface; and (C) the subsequent transfer of the phenols from the solid to the liquid [11]. In addition, another slow action would include several chemical reactions that have been reported to exert a certain influence on the extraction of phenolic compounds [32]. Out of all of them, the oxidation reactions are the most important as they involve both the compounds from the distillate, such as the acetaldehyde [33], and other phenolic compounds of the wood [9,34]. All these reactions modify the concentration of the phenolic compounds in the distillate as the aging process progresses. 

Based on these factors, the Peleg’s pseudo-second order model was selected as more being adequate to explain the kinetics of the phenolic compounds extractions that were performed for the purposes of this paper.

### 3.2. Kinetics of the Non-Sonicated Extractions at Different Temperatures

Once the Peleg’s pseudo-second order model was selected to fit the kinetics of the phenolic extractions, the kinetic constants, *K*_1_ and *K*_2_, were determined for each of the six extraction procedures that were carried out at different temperature levels, applying Equation (2). Then, the initial phenolic compound extraction rates and the theoretical TPI in equilibrium were calculated by applying Equations (3) and (4), respectively.

**Figure 3:**
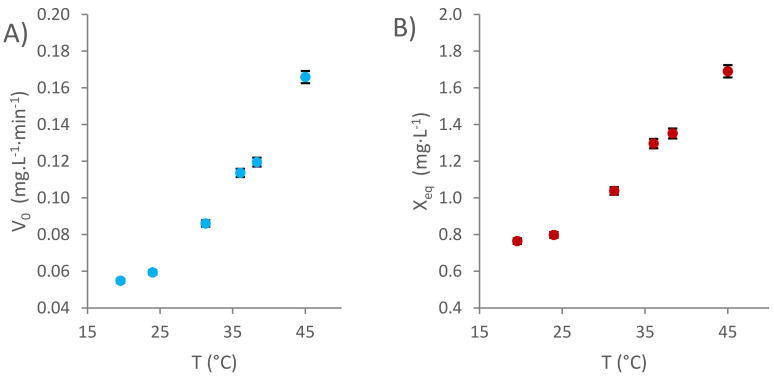
Kinetic parameters at six different temperature levels: (**A**) initial extraction rate and (**B**) theoretical TPI at equilibrium.

The initial extraction rates, expressed in mg·L^−1^·min^−1^ GAE, are plotted in Figure 3A. As can be observed, the initial extraction rate increases as the temperature rises from 19.56 °C to 45.01 °C. According to the previous studies, the effect on the extraction rates can be explained by two different factors: firstly, the solvent molecules are more likely to reach the necessary activation energy at higher temperatures [14]; secondly, these molecules move faster at higher temperatures, which favors the diffusion through the wood pores and, in turn, accelerates the diffusive extraction steps [11].

The theoretical TPI in the equilibrium values are calculated and plotted in Figure 3B. Such values increase as the temperature of the extraction media rises from the minimum to the maximum studied ones, probably due to the changes in the solubility of the phenolic compounds. Such a change has already been reported in the literature by several authors [35]. In this way, Noubigh et al. [36] stated that the solubility of various phenolic compounds, such as gallic, vanillic, syringic and protocatechuic acids, is correlated with the temperature following Van’t Hoff model. 

In order to quantify the acceleration of the extraction process caused by sonication, regression curves had to be determined by means of the Arrhenius equation. Thus, a correlation between the resulting kinetic constants and the temperatures at which those extractions were carried out could be established. 

**Figure 4:**
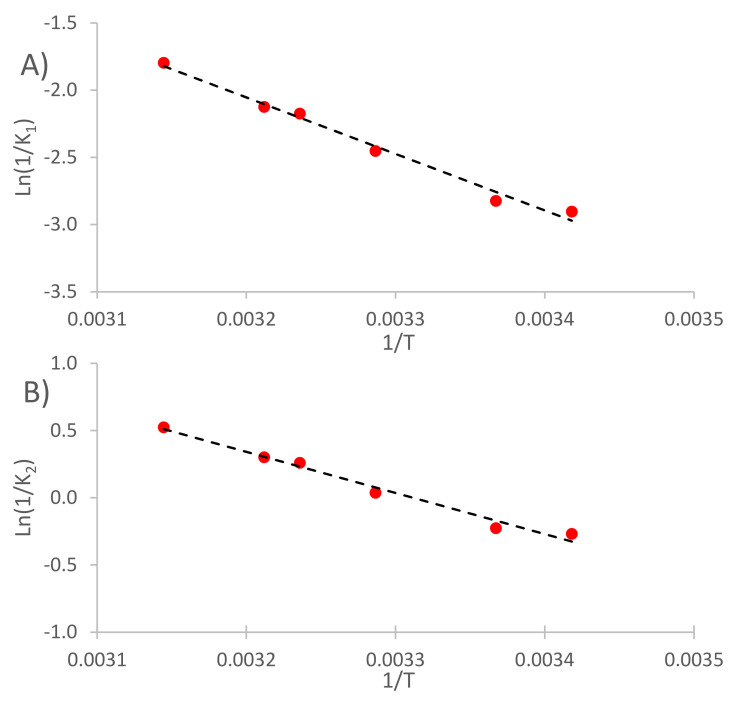
Arrhenius plots of the six non-sonicated extractions at different temperatures (NUS), (**A**) *K*_1_ constant and (**B**) *K*_2_ constant.

The Arrhenius plot for the constant *K*_1_ is shown in Figure 4A, returning a coefficient of determination R^2^ of 0.9868 and an activation energy of 34.98 KJ.mol^−1^. In addition, the Arrhenius plot using the constant *K*_2_ is shown in Figure 4B, obtaining an R^2^ value of 0.9820 and an activation energy of 25.46 KJ.mol^−1^. As *K*_1_ is related to the initial extraction rate and *K*_2_ is related to the TPI at equilibrium, these activation energy values allow us to believe that the initial and fast diffusion process had to overcome higher energy barriers to initiate the extraction, more than those that faced the slower diffusion processes. This result is in accordance with that obtained by Yang Tao et al. [16], concerning the extraction of phenolic compounds from grape marc, who reported that higher activation energy levels were required to start the fast diffusion process than those required for the slow diffusion process.

### 3.3. Effects of Ultrasound on the Kinetics of the Extraction Process

**Figure 5:**
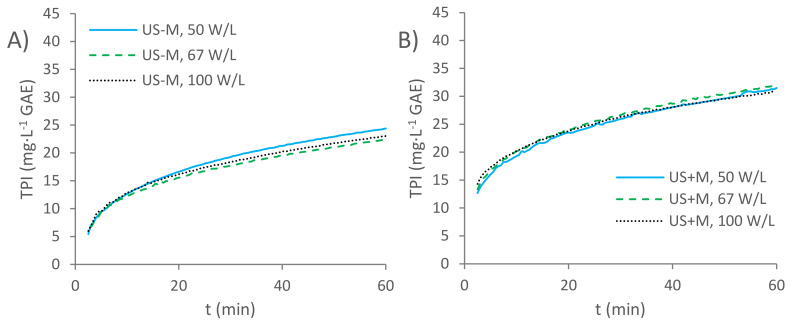
TPI vs. time plots at three increasing ultrasound power density levels: (**A**) non-pumped sonicated extractions (US-M) and (**B**) pumped sonicated extractions (US + M).

Once the Arrhenius plots were obtained for each of the kinetic constants, according to Peleg’s kinetic model, determining the kinetics of the extraction experiments that were carried out with sonication at different ultrasound power densities was the next step to be completed. The TPI vs. time curves obtained by the three extraction procedures with sonication, but without the movement of the distillate, were plotted, as shown in Figure 5A. 

The resulting TPI values at the initial times were similar for the three extraction experiments, where there were no statistical differences between them, after applying ANOVA at a 95% confidence level. On the other hand, they differed at the end of the extraction process: the TPI was lower (22.392 ± 0.201 mg·L^−1^ GAE) for the extraction carried out at 67 W·L^−1^, and higher for the extraction carried out at 50 W·L^−1^ (24.388 ± 0.293 mg·L^−1^ GAE). The extraction carried out under a sonication at 100 W·L^−1^ returned an intermediate value of 23.046 ± 0.369 mg·L^−1^ GAE. This tendency may be a consequence of the degradation of the phenolic compounds when submitted to high power ultrasounds that favor their oxidation and condensation reactions, or that may degrade them by disrupting or breaking their molecular bonds [9].

**Figure 6:**
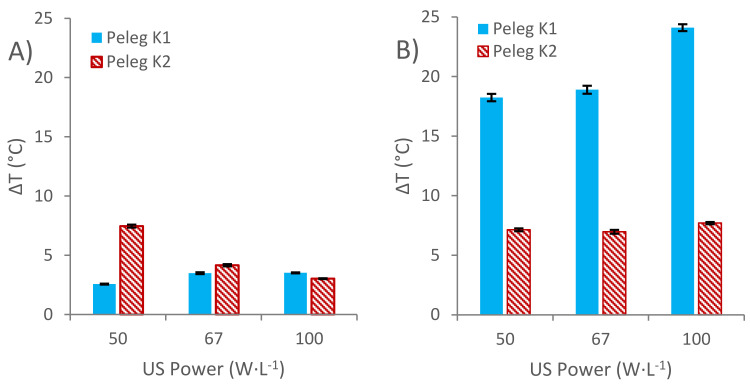
Temperature increments (ΔT) related to Peleg’s *K*_1_ and *K*_2_ constants at three different ultrasound power density levels: (**A**) non-pumped sonicated extractions (US-M) and (**B**) pumped sonicated extractions (US-M).

After the kinetic modeling was completed, the resulting *K*_1_ and *K*_2_ constants were interpolated into the Arrhenius plots that were previously shown (Figure 4A,B), in order to calculate the theoretical temperature at which each extraction should have been carried out without sonication for obtaining the same kinetics. Finally, by applying Equation (6), the fictional increments of the temperature produced by the sonication of the extraction media were obtained and plotted in Figure 6A, including the increments associated with the initial extraction rate (*K*_1_ constant) and the increments associated with the TPI at equilibrium (*K*_2_ constant).

The increments of temperatures corresponding to *K*_1_ when the extraction media was sonicated show an increasing tendency as the ultrasound’s power is increased from 50 W·L^−1^ to 67 W·L^−1^, while there were no statistical differences between 67 W·L^−1^ and 100 W·L^−1^. In this way, for 50 W·L^−1^, the temperature at which the extraction should have been carried out without sonication to obtain the same initial rate was determined as 2.57 ± 0.04 °C higher than the actual temperature of the distillate, while for 67 W·L^−1^ this increment was 3.49 ± 0.08 °C, and for 100 W·L^−1^ the difference in temperature was 3.52 ± 0.04 °C.

Taking these results into consideration, we can assume that higher ultrasound power levels are associated with higher initial rates in the extraction processes of phenolic compounds. Nevertheless, it seems that there is a certain ultrasound power level beyond which no significant extraction rate increase is achieved. The accelerating extraction effect seems to be produced by the large amount of energy that is released at localized points on the surface of the solid, due to the implosion of the cavitation bubbles formed by ultrasonic energy. When this large amount of energy is released, it overcomes the activation energy required by the extraction process and causes the solvent molecules to break the bonds between the wood and the phenolic compounds that can be found on its surface [14]. On the other hand, it seems that when ultrasound is applied at 67 W·L^−1^, the necessary activation energy to break the above-mentioned bonds is already applied to the system, and, therefore, an increment in ultrasound power does not create any further effect on the extraction rate. 

The increments in the temperature corresponding to *K*_2_ when the extraction media is sonicated, show that there is a decreasing tendency as the ultrasound power level is increased. For instance, when ultrasound is applied to the distillate at 50 W·L^−1^, the theoretical temperature at which the extraction should have been carried out without sonication to obtain the same TPI at equilibrium is 7.46 ± 0.13 °C higher than the actual one. On the other hand, when 67 W·L^−1^ ultrasound power is applied, the corresponding temperature increment decreases to just 4.16 ± 0.09 °C. Following this falling trend, when the ultrasound power is increased to 100 W·L^−1^, the increment in temperature is reduced to just 3.03 ± 0.04 °C.

According to these results, the final TPI generally increases with the sonication of the extraction media. This effect could be explained by the presence of free radicals generated by the sonolysis of the water that takes place when the water–ethanol mixture is sonicated. The hydroxyl and hydrogen peroxide generated by this phenomenon may induce oxidation reactions that would affect the equilibrium state of the phenolic compounds [4]. Thus, the falling trend of the theoretical temperature increment as the ultrasound power is increased could be explain by a greater degradation in the phenolic compounds submitted to higher power ultrasounds, which would disrupt and/or break their molecular bonds.

According to these results, it could be stated that ultrasounds increase both the initial phenolic’s extraction rate and the TPI at equilibrium, and that the temperature at which similar parameters would be obtained without any sonication is, at minimum, 2.5 °C higher to obtain the same initial rate, and around 3.0 °C higher to reach the same TPI. It can therefore be affirmed that the extraction acceleration effect of ultrasounds was proven and quantified. 

### 3.4. Synergic Effect Resulting from the Movement of the Pumped Distillate and the Application of Ultrasounds

Once the effects on the extraction of the phenolic compounds achieved by applying ultrasound energy were quantified, our research team considered that the movement of the pumped distillate through the wood chips while submitted to the ultrasound waves could have a synergic effect that should be studied [4]. In our experimental model, a continuous flow of the distillate was pumped from a glass tank and passed through a sonicated extraction chamber, where a certain amount of wood chips were submerged. Therefore, the ultrasound waves were only applied on the distillate while it passed through the wood chips. In this way, as explained above, the ultrasound waves accelerated the extraction of the phenolic compounds found in the wood chips, while, at the same time, the stress that phenols would suffer if submitted to continuous sonication was mitigated to some extent. 

The TPI vs. time curves for the extraction experiments in the presence of ultrasounds and the movement of the distillate through the wood can be observed in Figure 5B. The TPI values at different times were similar for the three extraction processes. At the initial time, the TPI was lower (with a value of 12.684 ± 0.152 mg·L^−1^ GAE) for the extraction carried out at 50 W·L^−1^, and higher for the extraction carried out at 100 W·L^−1^ (with a value of 14.324 ± 0.172 mg·L^−1^ GAE). Therefore, the extraction of phenols at the beginning of the extraction procedure seems to increase as the ultrasound power level is also increased. On the other hand, the final TPI values follow a different trend: the highest TPI value after completing the extraction process was obtained for the extraction that was carried out at 67 W·L^−1^, with a value of 32.004 ± 0.192 mg·L^−1^ GAE, while the lowest valued corresponded to the extraction carried out at 100 W W·L^−1^, with a value of 30.785 ± 0.185 mg·L^−1^ GAE.

The *K*_1_ and *K*_2_ constants obtained by applying Peleg’s model were interpolated into the Arrhenius plots previously shown (Figure 4A,B), and the temperature increments caused by the sonication of the extraction media are plotted in Figure 6B, where the increments corresponding to each of the three different ultrasound power levels applied, associated with the initial extraction rate (*K*_1_) and with the TPI at equilibrium (*K*_2_), can be seen.

With regard to *K*_1_, the increments of the temperature generated by the sonication of the extraction media show an increasing tendency as the ultrasound power level is increased. Thus, for 50 W·L^−1^, the temperature at which the non-sonicated extraction should have been carried to obtain the same initial rate is 18.24 ± 0.31 °C higher than the actual one. For 67 W·L^−1^, this increment is 18.9 ± 0.33 °C, and for 100 W·L^−1^ it is 24.11 ± 0.29 °C.

According to these results, higher powers of ultrasound produce higher initial rates for the extraction process of phenolic compounds from wood. This acceleration could be produced by the overcoming of the activation energy of the process, and by the improvement of the movement of the liquid’s molecules through the wood pores, thanks to the flowing pressure produced by the continuous pumping of the liquid through the reaction tank containing the wood chips. 

On the other hand, although this trend is in accordance with the trend that was observed in the experiments with sonication, but without distillate pumping, in this case, no restriction was registered with regard to the maximum initial rates. This could be explained by the synergy effect that takes place, as the best flow through the wood pores is combined with the effect of the implosive energy produced by cavitation bubbles, which would improve the diffusive extraction of the phenolic compounds found in the wood pores.

With regard to the temperature increments associated with *K*_2_, no statistical differences were observed between the improvement on the total phenolic index obtained from the process when carried out at 50 W·L^−1^ (with an increment of 7.14 ± 0.12 °C) and the extraction carried out at 67 W·L^−1^ (with an increment of 6.97 ± 0.16 °C), applying ANOVA at a 95% confidence level. On the other hand, the TPI obtained at 100 W·L^−1^ was equivalent to one that should be obtained at a theoretical temperature 7.70 ± 0.09 °C higher than the actual extraction temperature.

According to these results, the TPI at equilibrium increases as the sonication power is increased. As it was previously explained, the sonication of water–ethanol mixtures produces oxidative species that favor certain oxidation processes that may alter the extraction equilibriums of the phenolic compounds. On the other hand, this increasing extraction effect is the opposite to that registered for the extractions that were carried out with sonication, but where the distillate was not pumped through the system. 

This contrary behavior could be explained by the extraction process itself. In the pumped systems, the ultrasound waves were only applied to the extraction cells, i.e., the fluid was not constantly submitted to sonication. Thus, the continuous stress caused to the phenolic compounds by the constant sonication of the distillate would be reduced, and the phenolic compounds would not experience such a severe degradation as in non-pumped systems. 

According to these results, the synergic effect resulting from the pumping and sonication of the extraction media was proven to accelerate extraction processes: the temperature at which non-sonicated extractions should have been carried out is, at least, around 18.2 °C higher to obtain the same initial rate, and around 7.0 °C higher to reach the same TPI. 

### 3.5. Comparison between Sonicated Extraction in Pumped and Non-Pumped Systems

**Figure 7:**
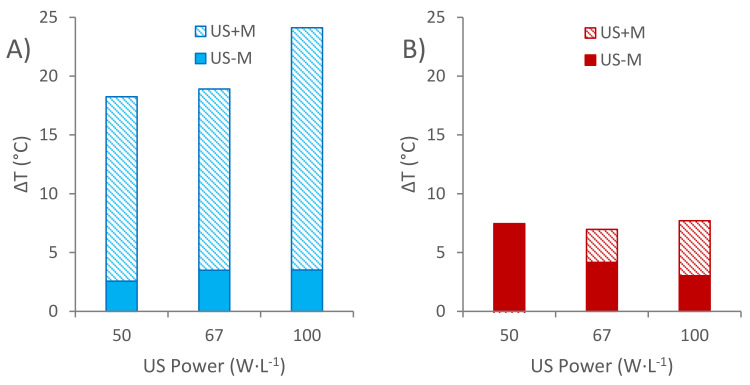
Theoretical temperature increment comparison related to Peleg’s (**A**) *K*_1_ and (**B**) *K*_2_ constants between non-pumped sonicated extractions (US-M) and pumped sonicated extractions (US + M).

In order to better visualize the comparison between the results obtained from non-pumped sonicated extractions (US-M) and the ones obtained from pumped sonicated extractions (US + M), all the temperature increments corresponding to the two studied kinetic constants were included and overlapped in Figure 7A,B (*K*_1_ and *K*_2_).

It can be seen in Figure 7A that the improvement of the initial extraction rate is substantially favored (around five-fold) by the synergy between the sonication of the media and the movement of the distillate through the wood chips, when compared to the sole sonication of the non-pumped distillate. On the other hand, as it is shown in Figure 7B, the increment of the temperature registered for the TPI at equilibrium is almost the same in each one of the experiments, where the distillate was pumped and sonicated, and it equals the temperature increment registered for the extraction experiments in the non-pumped systems where 50 W·L^−1^ ultrasound power was applied. 

## 4. Conclusions

In conclusion, the theoretical temperature required in non-sonicated systems to reach the same initial extraction rate that would be achieved with the use of sonication was determined as 2.5 °C higher. Furthermore, when the distillate was continuously pumped through the wood chips, the said theoretical temperature would be as much as 18.2 °C higher. With regard to the conditions to reach the TPI in equilibrium, non-sonicated systems would require a 3.0 °C higher temperature than the non-pumped sonicated systems, while in comparison to the temperature required for pumped sonicated systems, the temperature difference would be up to 7.0 °C. This paper proves that the phenolic extraction by alcoholic distillates from wood can benefit from the application of ultrasound. It also offers an approximation of the quantification of the acceleration that the sonication produces on the extraction process. Finally, the favorable synergy that results from pumping the distillate through the wood chips in a continuous flow is quantified.

These results are the first step for assuming the convenience of developing new accelerated aging procedures by applying ultrasound waves, and, when possible, designing pumped systems that allow spirit producers to either develop new products or improve their current products by shortening their aging time length requirements. 

## Figures and Tables

**Table 1:** Thermal extraction R^2^ values (NUS) by applying the two proposed kinetic models on the experiments’ TPI vs. time plots.

	Lagergren	Peleg
NUS, 20 °C	0.9377	0.9868
NUS, 25 °C	0.9360	0.9877
NUS, 35 °C	0.9309	0.9885
NUS, 40 °C	0.9313	0.9887
NUS, 45 °C	0.9216	0.9868
NUS, 50 °C	0.9042	0.9887

## Data Availability

Not applicable.
